# Long-Term Anti-Allodynic Effect of Immediate Pulsed Radiofrequency Modulation through Down-Regulation of Insulin-Like Growth Factor 2 in a Neuropathic Pain Model

**DOI:** 10.3390/ijms161126013

**Published:** 2015-11-13

**Authors:** Chun-Chang Yeh, Hsiao-Lun Sun, Chi-Jung Huang, Chih-Shung Wong, Chen-Hwan Cherng, Billy Keon Huh, Jinn-Shyan Wang, Chih-Cheng Chien

**Affiliations:** 1 Department of Chemistry, Fu-Jen Catholic University and Graduate Institute of Basic Medicine, Fu-Jen Catholic University, New Taipei City 24205, Taiwan; anes2yeh@gmail.com; 2 Department of Anesthesiology and Integrated Pain Management Center, Tri-Service General Hospital and National Defense Medical Center, Taipei 11490, Taiwan; w82556@gmail.com (C.-S.W.); cherng1018@yahoo.com.tw (C.-H.C.); 3 School of Medicine, Fu Jen Catholic University, New Taipei City 24205, Taiwan; 4 Department of Anesthesiology, Sijhih Cathay General Hospital, New Taipei City 22174, Taiwan; hlsun@cgh.org.tw; 5 Department of Medical Research, Cathay General Hospital, Taipei 10631, Taiwan; science.man2@gmail.com; 6 Department of Biochemistry, National Defense Medical Center, Taipei 11490, Taiwan; 7 Department of Anesthesiology, Cathay General Hospital, Taipei 10631, Taiwan; 8 Department of Pain Medicine, University of Texas MD Anderson Cancer Center, Houston, TX 77030, USA; BKHuh@mdanderson.org

**Keywords:** pulsed radiofrequency, spared nerve injury, extra-cellular signal-regulated kinase 1/2, Insulin-like growth factor II, gene ontology annotations

## Abstract

Pulsed radiofrequency (PRF) is effective in the treatment of neuropathic pain in clinical practice. Its application to sites proximal to nerve injury can inhibit the activity of extra-cellular signal-regulated kinase (ERK) for up to 28 days. The spared nerve injury (SNI)+ immPRF group (immediate exposure to PRF for 6 min after SNI) exhibited a greater anti-allodynic effect compared with the control group (SNI alone) or the SNI + postPRF group (application of PRF for 6 min on the 14th day after SNI). Insulin-like growth factor 2 (IGF2) was selected using microarray assays and according to web-based gene ontology annotations in the SNI + immPRF group. An increase in IGF2 and activation of ERK1/2 were attenuated by the immPRF treatment compared with an SNI control group. Using immunofluorescent staining, we detected co-localized phosphorylated ERK1/2 and IGF2 in the dorsal horn regions of rats from the SNI group, where the IGF2 protein predominantly arose in CD11b- or NeuN-positive cells, whereas IGF2 immunoreactivity was not detected in the SNI + immPRF group. Taken together, these results suggest that PRF treatment immediately after nerve injury significantly inhibited the development of neuropathic pain with a lasting effect, most likely through IGF2 down-regulation and the inhibition of ERK1/2 activity primarily in microglial cells.

## 1. Introduction

Pulsed radiofrequency (PRF) is effective for some types of chronic intractable pain [1,2,3,4,5,6,7,8,9]. Unlike conventional high-temperature radiofrequency, PRF can deliver a brief high-frequency electrical stimulation near a dorsal root ganglion (DRG) or a sensory nerve without significant nerve damage [10].

PRF application adjacent to a DRG or sensory nerve might change biological activity of synaptic transmission, cell morphology, or c-Fos expression in the superficial dorsal horn of the spinal cord, with a trivial effect on nerve tissue [11,12,13]. However, the mechanism of PRF action remains elusive. Evidence from the existing literature indicates that extra-cellular signal-regulated kinase (ERK) plays a critical role in regulating inflammatory responses and neuropathic pain [14,15]. Otsubo *et al.* showed that ERK knockout mice have reduced pain sensitization after formalin stimulation or partial sciatic nerve ligation [16]. Low-voltage PRF may attenuate mechanical allodynia and thermal hyperalgesia in a rat model of neuropathic pain produced by spinal nerve ligation (SNL) by affecting the phosphorylation of ERK [17]. Immediate PRF application to sites proximal to nerve injury significantly inhibited the development of neuropathic pain, accompanied by inhibited ERK activation in rats after spared nerve injury (SNI) [18]. Therefore, the inhibition of ERK activation might be a novel target for the treatment of neuropathic pain [19,20,21].

Various durations of anti-allodynia effects have been observed following PRF application after nerve injury, but most PRF applications were applied a considerable time after the development of neuropathic pain [22,23,24]. We were among the first to apply PRF immediately to alleviate SNI-induced neuropathic pain and found that immediate PRF of 60 V for 6 min had anti-nociceptive effects for up to 28 days [18].

To gain a better understanding of the potential molecular mechanism underlying alleviation of pathological pain by PRF, we herein employ a rat model of spared nerve injury (SNI) to investigate the impact of PRF on the modulation of pain-regulatory genes after nerve injury. Potential anti-allodynic effects were evaluated after PRF treatments applied at two different times: for 6 min immediately after SNI (SNI + immPRF), and for 6 min on the 14th day after SNI (SNI + postPRF). The resulting differences in rat spinal cords were assessed at a molecular level using whole-genome microarrays. Subsequently, gene ontology (GO) annotations and gene expression analyses were also conducted. Dorsal horns of rats with SNI or SNI + immPRF treatment were stained immunofluorescently to localize the distributions of target proteins *in vivo*.

## 2. Results

### 2.1. Anti-Allodynic Effect of Immediate PRF Treatment

**Figure 1:**
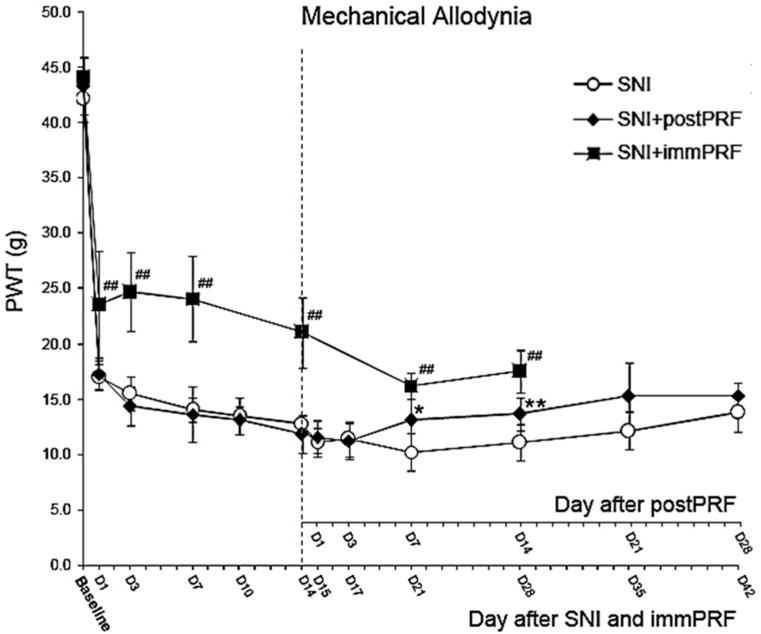
Comparison of PRF treatments applied at two different times: immediately after SNI (SNI + immPRF), and on the 14th day after SNI (SNI + postPRF), in reducing mechanical allodynia in spared nerve injured (SNI) rats. SNI + immPRF group, with immediate PRF-60V (6 min) following SNI; SNI + postPRF group, with PRF-60V (6 min) on the 14th day after SNI; PRF, pulsed radiofrequency; PWT, Paw withdrawal threshold. D1 to D42 meant day 1 to day 42 after treatment. * *p* < 0.05, SNI group compared with SNI + postPRF group. ** *p* < 0.01, SNI group compared with SNI + postPRF group. ^##^ *p* < 0.01, SNI group compared with the SNI + immPRF group.

Immediate PRF treatment (the SNI + immPRF group; black squares in Figure 1) provided a significantly better anti-allodynic effect compared with delayed PRF treatment (the SNI + postPRF group; black diamonds in Figure 1). As shown in Figure 1, the anti-allodynic effect of immPRF treatment lasted throughout the entire observation period of 28 days. However, rats from the SNI + postPRF group showed a higher paw-withdrawal threshold only on Day 7 and Day 14 post-treatment (*i.e.*, Day 21 and Day 28 post-injury), but no obvious anti-allodynic effect on Days 1, 3, 21, or 28 post-treatment.

### 2.2. Bioinformatic Analyses from Oligonucleotide Microarray Hybridization and Gene Validation

**Figure 2:**
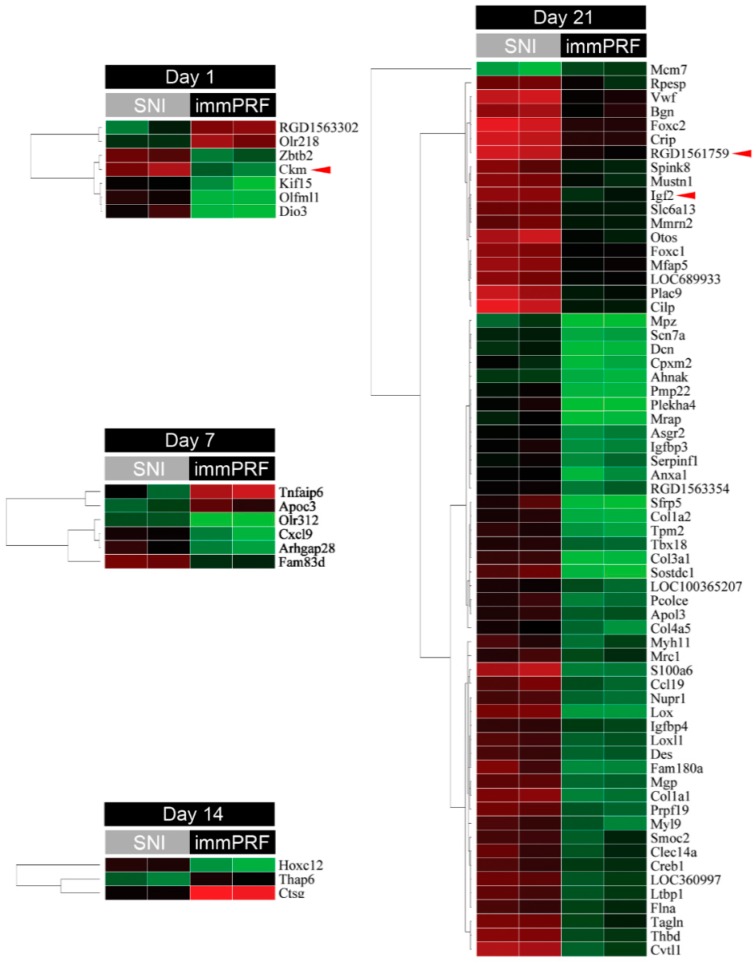
Significant genes in immediate PRF treatment. Comparison of gene expression in rats from the SNI and SNI + immPRF groups at various times. A total of 80 genes with significant differential expression (>2-fold, *p* < 0.05) were found from the microarray hybridizations. Three genes (*Ckm*, *RGD1591759*, and *Igf2*; indicated by red triangle) with ERK- or MAPK-related GO terms were separated according to the Web-based gene ontology annotations (http://david.abcc.ncifcrf.gov).

**Figure 3:**
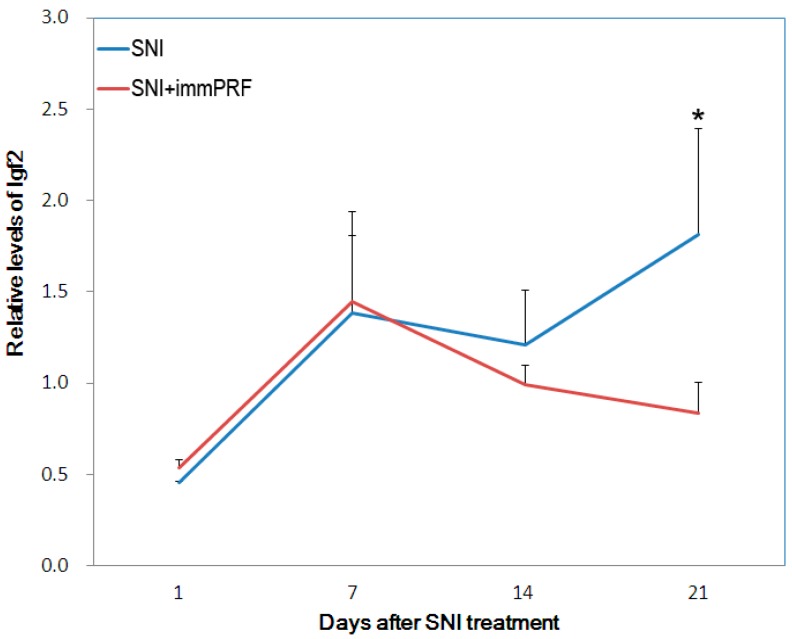
Validation of Igf2 expression by quantitative real-time PCR. Relative expression of Igf2 in dorsal horns (L4–L6) was quantified and normalized to individual expression of GAPDH. Each quantitation was independently triplicated. SNI, spared nerve injury; PRF, pulsed radiofrequency. Blue line, SNI group; red line, SNI + immPRF group. * *p* < 0.05.

From the Agilent Rat Genome Oligo 4 × 44 K Microarrays, 80 genes with significant differential expression were found in SNI-immPRF-treated rats (>2-fold, *p* < 0.05) (Figure 2). As listed in Table 1, four enriched GO terms (extra-cellular matrix, extra-cellular region part, extra-cellular region, and extra-cellular matrix part) were filtered according to Parent–Child–Union—Bonferroni criteria (adjusted *p* < 0.001). Three genes (for *Ckm*, *RGD1591759*, and *Igf2*) were selected from the 80 genes mentioned because of their relationships with ERK and mitogen-activated protein kinase (MAPK) pathways found using an online GO database [18]. Among them, IGF2 was chosen as our target, because IGF2 was the only one molecule that was acquired by cross-matching the two databases. By comparison with the up-regulated IGF2 in SNI rats, its levels of expression in SNI-immPRF rats at Day 21 were close to those in rats at Day 0 (Figure 2). This differential expression of IGF2 was confirmed by real-time PCR. As shown in Figure 3, the levels of IGF2 mRNA were gradually decreased in the SNI-immPRF rats after Day 7 and exhibited a significant difference on Day 21.

**Table 1:** Four enriched GO terms were filtered according to Parent–Child–Union—Bonferroni criteria (adjusted *p* < 0.001).

GO Term	GO ID	Subontology	Genes	Pop. %	Study %	adj. *P*
extracellular matrix	GO:0031012	Cellular Component	Bgn, Cilp, Col1a1, Col1a2, Col3a1, Col4a5, Cpxm2, Ctsg, Dcn, Lox, Loxl1, Ltbp1, Mfap5, Mgp, Pcolce, Rpesp, Serpinf1, Smoc2, Vwf	2.18%	28.36%	<0.001
extracellular region part	GO:0044421	Cellular Component	Anxa1, Apoc3, Bgn, Ccl19, Cilp, Col1a1, Col1a2, Col3a1, Col4a5, Cpxm2, Ctsg, Cxcl9, Dcn, Igf2, Igfbp3, Igfbp4, Lox, Ltbp1, Mfap5, Mgp, Pcolce, Serpinf1, Smoc2, Sostdc1, Vwf	5.36%	37.31%	<0.001
extracellular region	GO:0005576	Cellular Component	Anxa1, Apoc3, Apol3, Bgn, Ccl19, Cilp, Col1a1, Col1a2, Col3a1, Col4a5, Cpxm2, Ctsg, Cxcl9, Dcn, Igf2, Igfbp3, Igfbp4, Lox, Ltbp1, Mfap5, Mgp, Olfml1, Otos, Pcolce, Serpinf1, Sfrp5, Smoc2, Sostdc1, Vwf	8.48%	44.78%	<0.001
extracellular matrix part	GO:0044420	Cellular Component	Cilp, Col1a1, Col1a2, Col3a1, Col4a5, Dcn, Lox, Mfap5, Smoc2	0.90%	13.43%	<0.001

### 2.3. Determination of ERK1/2 Phosphorylation in the Presence of IGF2

**Figure 4:**
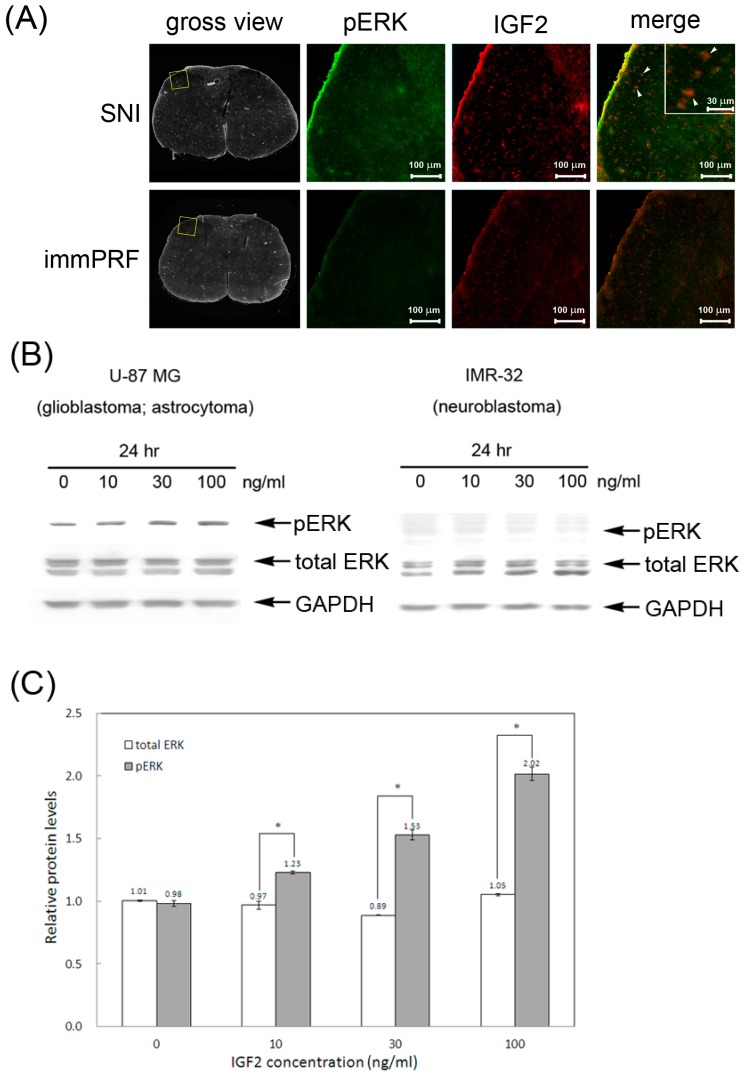
Correlation of phosphorylated ERK and IGF2. (**A**) *In situ* immunofluorescent staining of phosphorylated ERK and IGF2 in spinal cords of rats after SNI or SNI + immPRF treatment. The blank square in the gross anatomical views indicates the dorsal horn region used to illustrate the positive immunoreactive signals indicating phosphorylated ERK (pERK, green) and IGF2 (red). The gross views were taken by visible light. White arrowheads show the merged pERK and IGF2 signals. Scale bars = 100 μm; (**B**) Immunodetection of total and phosphorylated ERK in U-87 MG and IMR-32 cells. The astrocytoma cells, U-87 MG (HTB-14), and neuroblastoma cells, IMR-32, were treated with various amounts of IGF2 (from 0 to 100 ng/mL). The total ERK protein and its phosphorylated form were detected with anti-ERK and anti-pERK antibodies, respectively. GAPDH was used as an internal control; (**C**) Relative quantitation of total and phosphorylated ERK of U-87 MG cells. The relative protein levels were determined by normalizing their expression to that of GAPDH through densitometric analyses. An independent *t* test was conducted to analyze two or three independent experiments. pERK, phosphorylated ERK. Blank bar, total ERK; grey bar, pERK. * *p* < 0.05.

**Figure 5:**
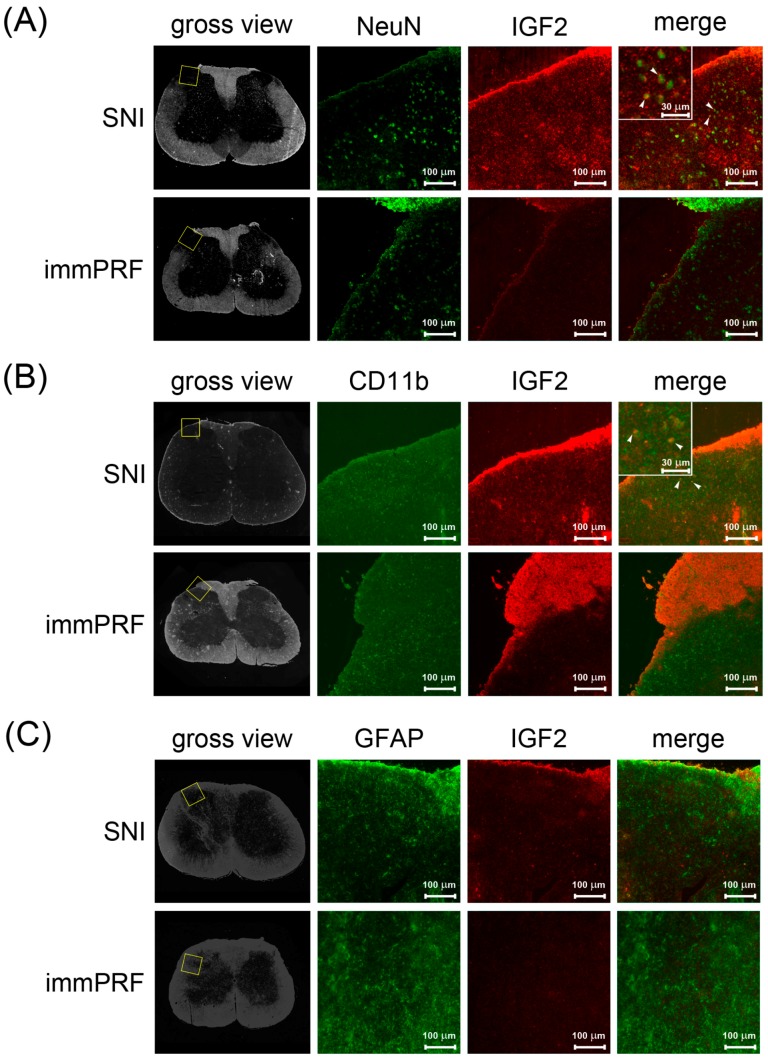
Identification of IGF2-expressing cells in the spinal cords of rats with SNI or SNI + immPRF treatment. (**A**) *In situ* immunofluorescent staining of NeuN and IGF2; (**B**) *in situ* immunofluorescent staining of Cd11b and IGF2; and (**C**) *In situ* immunofluorescent staining of GFAP and IGF2. The blank square in the gross anatomical views indicates the dorsal horn region used to illustrate the positive signals. All the gross views were taken by visible light. White arrowheads indicate merged signals. Green: NeuN, Cd11b, or GFAP. Red: IGF2. Scale bars = 100 μm.

To establish whether the repression of IGF2 plays a role in PRF-induced anti-allodynic effects, we analyzed the expression of IGF2 in the left L4–L6 dorsal horns of rats under different treatments (SNI alone and SNI + immPRF). Meanwhile, we used the phosphorylated ERK1/2 in response to the nerve injury to evaluate allodynia as described by others [17,18]. Figure 4A shows the immunofluorescent images of IGF2 and phosphorylated ERK1/2 immunoreactivity on sections taken from the left dorsal horn region of rats with different treatments on Day 21. We detected the co-localization of IGF2 and active ERK1/2 in the left dorsal horn region of rats from the SNI group (the upper panel of Figure 4A). At the same time, phosphorylated ERK1/2 and IGF2 were rarely observed in rats from the SNI + immPRF group (the lower panel of Figure 4A). Moreover, using an *in vitro* approach, we further investigate whether exogenous IGF2 could induce ERK1/2 phosphorylation and what kind of cells involve in the process of neuropathic pain development. We employed two brain tumor cell lines, U-87MG and IMR-32, which have differential immunoreactivity (Figure 4B). IMR-32 cells showed no obvious phosphorylated ERK1/2 after IGF2 treatment in a dose-dependent manner, whereas the relative amount of phosphorylated ERK1/2 increased significantly from 0.98 to 2.02, while the total levels of ERK1/2 did not change significantly in U-87MG cells (from 1.01 to 1.05) treated with IGF2 for 24 h (Figure 4C). To determine whether IGF2-immunoreactive cells in spinal dorsal horn are neurons, microglia or astrocytes, we performed double immunofluorescent staining of IGF2 with several cell-specific markers: NeuN (neurons), CD-11b (microglia), and GFAP (astrocytes) (Figure 5). The fluorescent signal of IGF2 immunoreactivity was highest in the NeuN- and CD11b-immunoreactive-positive cells (Figure 5A for NeuN and Figure 5B for CD11b). However, in the left dorsal horn of rats, IGF2 was not detected in GFAP-immunoreactive-positive cells in rats from the SNI group (Figure 5C).

## 3. Discussion

PRF therapy is a frequently used option in the clinical treatment of various types of pain [25,26,27]. We aimed to investigate the impact of PRF on the modulation of genes regulating pain. For the first time to our knowledge, this study has shown that a single immPRF-60V treatment provided a better anti-allodynic effect than postPRF-60V treatment when examining mechanical allodynia. It is very crucial to understand the underlying etiology of neuropathic pain in different models prior to clinical application in humans. Therefore, in translating our findings into future clinical treatment strategies to prevent the development of neuropathic pain, clinical research-based investigations should be performed to determine the difference in the anti-allodynic effect between immediate and delayed PRF treatment after acute nerve injury.

As reported by Lin *et al.* using a rat SNL model, the analgesic effect of low-voltage (0.25 to 0.3 V) early PRF treatment applied to the L5 DRG after nerve injury may be attributed to an inhibitory effect on ERK1/2 activation in dorsal horn cells at 30 min and on Day 3 [17]. Similarly, in our previous study, we discovered that immediate PRF-45V and PRF-60V treatments after SNI inhibited the activation of (ERK1/2) in the ipsilateral spinal dorsal horn of SNI rats on Day 28. In the present study, we demonstrated that immPRF-60V inhibited mechanical allodynia for much longer than postPRF-60V. The modulation of immPRF treatment should imply a unique molecular mechanisms contributing to long-term anti-allodynia.

### 3.1. Ckm, RGD1561759, and IGF2 Decreased in Spared-Nerve-Injured Rats with immPRF Treatment

ERK1/2 and MAPK activation in the spinal cord are correlated with pain through their regulation of many molecules [28]. Genome-wide expression and bioinformatics analyses have been used to profile the complex syndrome of pain and provide new insights into related molecular mechanisms [29,30,31,32]. We filtered three molecules from among the 80 genes with significant differential expression in the dorsal horn cells of rats that received immediate PRF-60V treatment. A well-known protein kinase, Ckm, and a putative protein kinase, RGD1561759, were down-regulated in immPRF-treated rats. Although human CKM (NM_001824) is found in molecularly abnormal malignant gliomas [33], Ckm and RGD1561759 have rarely been discussed in the context of neuronal disorders. By contrast, human IGF2 is one of three hormones with an insulin-like structure, and enhanced IGF2 signaling might be associated with adult neurogenesis [34,35].

### 3.2. Human IGF2 Induced ERK1/2 Phosphorylation during SNI-Induced Neuropathic Pain and Showed Predominantly in Microglial Cells

The present study showed that only IGF2 was further cross-matched using two enriched GO terms (GO:0044421, extra-cellular region part; GO:0005576, extra-cellular region) [36]. Exogenous IGF2 restores synapse density and promotes spine maturation [37]. This process depends on the IGF2-mediated MAPK/ERK pathway and increased levels of IGF2 coincided with elevated ERK1/2 phosphorylation [38]. Similarly, our data showed that IGF2 was up-regulated accompanied by ERK1/2 activation in the dorsal horn regions in rats with SNI. However, this up-regulation and activation were both attenuated when the rats underwent immPRF treatment (Figure 4A). In addition, we demonstrated that exogenous IGF2 induced ERK1/2 phosphorylation in glioblastoma cells (U-87MG), but not in neuroblastoma cells (IMR-32) (Figure 4B).

### 3.3. Down-Regulation of IGF2 May Be the Result of Immediate PRF

Neuropathic pain can induce central and peripheral sensitization. Central sensitization has been suggested to be a phenomenon similar to long-term potentiation, which is associated with learning and memory [39,40]. Now, new evidence from experimental studies has suggested that IGF2 play a role in neural plasticity, learning and memory [37,41]. The up-regulation of IGF2 in glial cells may result in inflammatory responses in humans [42], and its activation may contribute to mechanical allodynia [43]. In the present study, we demonstrated that decreased IGF2 may down-regulate the inflammatory response by reducing the activated form of ERK1/2. A long-term beneficial effect of PRF therapyin clinical treatment of neuropathic pain has been reported [5,44]. In our rat model of neuropathic pain, the application of immediate PRF at a site proximal to nerve injury appeared to provide effective analgesia via the inactivation of a MAPK/ERK pathway in neuronal and microglial cells; this inactivation is likely regulated by IGF2. The underlying mechanism most probably involves the spinal neurons and microglial cells, and IGF2 appears to play a crucial role in the immPRF-induced reversal of mechanical allodynia via the inhibition of ERK1/2 phosphorylation.

In the SNI group, we found that phosphorylated ERK1/2 co-localized with IGF2 (Figure 4A), where IGF2 immunoreactivity was mainly found in spinal microglial and neuronal cells (Figure 5A,B). By contrast, we found down-regulation of IGF2 after the application of immPRF treatment. Results from our study indicated that immPRF treatment after nerve injury mediated inhibition of SNI-induced IGF2, and ERK1/2 activation via spinal microglia may be a possible mechanism underlying its inhibitory action on SNI-induced neuropathic pain.

Gene expression profiling studies propose that persistent intractable pain is related to certain neuronal genes, along with genes related to immune cell responses, such as microglial activation [45,46]. In a model of neuropathic pain, microglial cells are forcibly manifested as early as one day, and up to 84 days after peripheral nerve injury, both at the spinal level and in the dorsal root ganglia [47,48]. Zhuang *et al.* indicated that the intrathecal administration of an ERK inhibitor suppressed spinal ERK activation, thus reversing mechanical allodynia, suggesting that astrocytic ERK plays a crucial role in the maintenance of neuropathic pain and astrocytes are possibly responsible for maintenance of pain [49,50]. By contrast, Deumens *et al.* demonstrated that three months after nerve injury, the amount of activated astrocytes is in inverse correlation with nociceptive hypersensitivity [51]. Our study investigated the impact of PRF on the modulation of pain-regulatory genes and ERK activity after nerve injury. Through using whole-genome microarrays, GO annotations, and gene expression analyses, IGF2 was detected and its involvement was confirmed by real-time PCR at 21 days after immPRF treatment by comparison with a SNI control group. We found that IGF2 immunoreactivity was mostly located in the spinal microglial and neuronal cells at 21 days after nerve injury and immPRF treatment suppressed its expression in spinal microglial and neuronal cells. To shed light on the mechanisms underlying these results, it is worth considering a few points. First, the immPRF treatment may suppress the initial cascade through pro-inflammatory cytokines after nerve injury and further reduce neuroinflammation to achieve a long-term anti-allodynic effect through the modulation of microglial and neuronal cells; Second, the immPRF treatment produced a long-term anti-allodynic effect by down-regulation of IGF2 and ERK activity through microglial and neuronal cells; Third, another explanation for these observations is that a small number of astrocytes still play a major role in the development of persistent pain. Previous study has reported that rats with a higher level of neuropathic pain revealed a lower intensity of GFAP expression in substantia gelatinosa at 12 weeks after nerve injury [51]. In our study, perhaps IGF2 was not obviously detected in the GFAP-positive cells in the SNI group because astroglial responses after peripheral nerve injury probably changed from a pro-nociceptive phenotype to an anti-nociceptive phenotype; Finally, our results reveal that immPRF treatment provides neuromodulation with a long-term anti-allodynic effect mainly through spinal microglia to inhibit SNI-induced IGF2 and ERK1/2 activation.

## 4. Materials and Methods

### 4.1. Rats, Neuropathic Pain Model

After approval by the Animal Care and Use Committee of the National Defense Medical Center, Taiwan, this experiment was performed according to the guidelines of the National Institutes of Health Guide for the Care and Use of Laboratory Animals (NIH Publication No. 80–23, revised in 1996). Male Wistar rats (BioLasco, Taipei, Taiwan), with weight of 200–250 g, were individually taken care of, with soft bedding on a 12 h cycle per day and night. They were free to obtain water and food at any time in the same circumstances for 7 days, for adaptation, prior to SNI surgery. The number of animals used and their suffering were minimized asleast as possible.

A model of neuropathic pain was produced using SNI, as described by Decosterd and Woolf [52], and single PRF was applied to rats in the various groups. Briefly, SNI was performed under 1.5% isoflurane (Halocarbon, River Edge, NJ, USA) anesthesia. A 2–4 mm section of ligated nerve from the common peroneal and tibial branches of the left sciatic nerve was removed, while the sural nerve was left intact. The rats were randomly assigned into three groups (*n* = 7 for each group): (i) an SNI group; (ii) an SNI + immPRF group; and (iii) an SNI + postPRF group. PRF was performed by an electrocautery disk, in a right decubitus position, connected to the PRF generator (NeuroTherm, NT1000, Surrey, UK). The 5 mm active tip electrode (NeuroTherm 22 GA) was put vertically adjacent to left sciatic nerve, 0.3–0.4 cm proximal to the injury site immediately after SNI surgery (*i.e.*, SNI + immPRF group) or on the 14th day after SNI surgery (*i.e.*, SNI + postPRF group). The PRF treatment (500 kHz) with an output of 60 V, or without output (*i.e.*, SNI group) was delivered at a rate of 2 Hz, 2 bursts per second and 20 milliseconds duration for 6 min with a temperature below 42 °C. The SNI group was used as control with placement of the electrode to 0.3–0.4 cm proximal to injury site, which was identical to those in PRF treatment groups, but without electric current applied. After the procedure, the skin incision was closed with 4-O silk sutures, and the animal was left alone to recover from anesthesia. Throughout 28 days, dynamic plantar aesthesiometry (DPA, Ugo Basile, Comerio, Italy) was used to evaluate mechanical allodynia.

### 4.2. Total RNA Extraction and Microarray Hybridization

In accordance with the manufacturer’s protocol (Qiagen, Venlo, The Netherlands), total RNA from samples of the left L4–L6 dorsal horns (which were collected in the presence of RNAlater reagent (Life Technologies, Carlsbad, CA, USA) was separately extracted with an RNeasy Mini Kit. Before microarray hybridization, total RNA extracted was quantified using a NanoDrop ND-1000 spectrophotometer (Thermo Fisher Scientific, Waltham, MA, USA) and a Bioanalyzer RNA Nano 6000 chip (Agilent Technologies, Santa Clara, CA, USA). Subsequently, 0.2 μg of the RNA was amplified into cRNA and labeled with Cy3-deoxycytidine triphosphate via an *in vitro* transcription process using a Low Input Quick-Amp Labeling Kit (Agilent Technologies). Cy3-labeled cRNA (0.6 μg) was sliced to an average length of about 50–100 nucleotides through culture growth with a fragmentation buffer at 60 °C for 30 min, followed by the differently fragmented cRNA (baseline and Day 21). The samples were separately hybridized by utilizing a Rat Genome Oligo 4 × 44 K Microarray of Agilent Technologies at a setting of 65 °C for 17 h. According to Agilent’s standard protocols, the microarrays were scanned by applying an Agilent Microarray Scanner at 535 nm, after washing and blow-drying.

### 4.3. Bioinformatics Analysis of Rat Whole-Genome Microarrays

We examined the scanned images with the Agilent Feature Extraction software (version 10.5.1.1). To quantify the signal and background intensity for each image, we analyzed the features and normalized the software. After the global normalization, low-intensity (<70) probes were excluded from further analysis. The relative levels (RLs) of expression of each gene in the SNI + immPRF group were assessed by comparing them with the level of expression of the same gene in the SNI group. Genes that were expressed differently in response to immPRF were selected based on a *t* test (RL > 2 or < 0.5-fold, *p* < 0.05). Furthermore, the genes of interest were ontologized by annotating and cross-matching candidates involved in the mitogen-activated protein kinase (MAPK)/ERK pathway according to Web-based GO annotations (http://david.abcc.ncifcrf.gov). To determine the GO-enriched terms, significant candidates were analyzed using an enrichment assay from the Ontologizer software (version 2.1; Charité Universitätsmedizin, Berlin, Germany).

### 4.4. Reverse Transcription and Validation of Rat IGF2 Expression

To confirm gene expression in response to PRF treatment, 1 μg of total RNA from the left L4–L6 dorsal horns were converted to single-stranded cDNA with the application of an ABI Reverse Transcriptase kit (Life Technologies).The mRNA expression of IGF2 was validated by quantitative real-time PCR (qPCR) through a universal probe library system (forward primer, CGCTTCAGTTTGTCTGTTCG; reverse primer, GCAGCACTCTTCCACGATG; UPL probe No. 40; Roche Diagnostics, Mannheim, Germany) in a LightCycler 1.5 Real-Time PCR system (Roche Applied Sciences, Mannheim, Germany). The RLs of target mRNA in various samples were normalized to that of glyceraldehyde 3-phosphate dehydrogenase (GAPDH, NM_017008) through a TaqMan probe-based gene expression analysis system (Rn01775763_g1; Life Technologies).

### 4.5. Glioblastoma Cells and Neuroblastoma Cells

The glioblastoma cell line U-87 MG (ATCC HTB-14) and the neuroblastoma cell line IMR-32 (ATCC CCL-127) were purchased from the American Type Culture Collection (ATCC, USA). Their clinical status and culture conditions were referenced on the ATCC website (www.atcc.org). Cells (1.25 × 10^5^ cells/well) were cultured in 6-well plates with medium containing 10% FBS for 48 h for IMR-32 and for 24 h for U-87 MG. Both cell lines were then treated with a serum-free medium for different times (24 h for IMR-32 and 48 h for U-87MG) in the absence or presence of the recombinant human insulin-like growth factor 2 (IGF2; I2526, Sigma, St. Louis, MO, USA) at indicated concentrations for a final 24 h incubation.

### 4.6. Immunofluorescent Staining of IGF2, Phosphorylated ERK, Glial Fibrillary Acidic Protein (GFAP), NeuN, and CD11b in the Dorsal Horn Regions of Rats

Ten-micrometer sections from rat L4–L6 spinal cords were fixed in 4% paraformaldehyde for 10 min and permeabilized with 0.1% Triton X-100 in phosphate-buffered saline for 35 min. Endogenous peroxidase activity was quenched using 3% H_2_O_2_ for 30 min and then non-specific binding sites were blocked by 30 min incubation in blocking solution (Vector Laboratories, Burlingame, CA, USA) at room temperature. Subsequently, the slides were incubated overnight in goat anti-IGF2 antibody (1:100; Santa Cruz Biotechnology, Dallas, TX, USA, sc-7435) at 4 °C and then for 1 h in HRP-conjugated antibody at room temperature. The IGF2 immunoreactive signal was developed using a Cy-3 Tyramide Signal Amplification (TSA) System (PerkinElmer, Waltham, MA, USA) according to the manufacturer’s instructions. For double staining, slides were then heated in a microwave oven to remove IGF2 antibody, as described by Toth and Mezey with minor modifications [53]. Briefly, the slides were stained using mouse anti-pERK (1:100; Santa Cruz Biotechnology, sc-7383), mouse anti-GFAP (1:100; EMD Millipore, Billerica, MA, USA, MAB3402), anti-NeuN (1:25; EMD Millipore, MAB377), and mouseanti-CD11b (1:100; Abcam, Cambridge, UK, ab75476) antibodies. Finally, all signals from double staining were developed using a fluorescein isothiocyanate TSA system.

### 4.7. Western Blotting of IGF2-Dependent Phosphorylated ERK in Human Neuronal Cells

Total protein of the cultured cells was extracted using a Pro-Prep protein extraction solution (iNtRON, Seoul, Korea) with a PhosSTOP Phosphatase Inhibitor (Roche Diagnostics, Mannheim, Germany). The concentration of the protein was then determined by utilizing the Bradford assay (Bio-Rad Laboratories, Hercules, CA, USA), and then 20 μg of protein was separated onto 12% sodium dodecyl sulfate polyacrylamide gel electrophoresis. Total ERK protein and its phosphorylated form blotted on a polyvinylidene difluoride membrane were respectively detected using a rabbit anti-ERK antibody (sc-93; Santa Cruz Biotechnology, Dallas, TX, USA) and the same anti-pERK antibody (both at a 1:500 dilution) as previously described. A specific secondary antibody from a Vectastain ABC-AmP kit (Vector Laboratories) was further hybridized at a 1:1000 dilution to determine the target proteins. GAPDH was used as an internal control. Protein bands were quantified by densitometric analysis using image processing and FluorChem FC2 software (Cell Biosciences, Santa Clara, CA, USA). The relative protein levels were calculated and determined by normalizing their expression to that of the GAPDH internal control.

### 4.8. Statistical Analysis

The SPSS software (version 14.0, SPSS Inc., Chicago, IL, USA) was used for the statistical tests. A one-way analysis of variance followed by a Bonferroni test was used for intergroup comparisons of variants with normal distribution values. A one-way variance analysis followed by a *post-hoc* analysis, the Bonferroni test, was used for intergroup comparisons of variants with normal distribution values. The Kruskal–Wallis test was applied to variants without a normal distribution. As described previously, an independent *t* test was conducted for analyzing the results of the microarray assays, qPCR, and Western blotting. For all the above tests, statistical significance was set at *p* < 0.05.

## 5. Conclusions

At present, the role of IGF2 in neuropathic pain is largely unknown. Results of the present study bring to light new insights into the potential function of IGF2 in nerve injury-induced pathological pain. ImmPRF treatment after nerve injury provided a significant inhibition of mechanical allodynia, mainly through the microglial pathway. In addition to reducing phosphorylation of ERK1/2, the anti-allodynic effect of immPRF modulation can also be attributed to a down-regulation of IGF2, primarily by microglial cells in the spinal dorsal horn.
